# Employing Metagenomics Capture targeted next-generation sequencing for the etiological diagnosis of bloodstream infections

**DOI:** 10.3389/fcimb.2026.1905007

**Published:** 2026-08-18

**Authors:** Chuyu Lao, Zihuan Li, Guanwen Lin, Congrong Li, Yuqi Wang

**Affiliations:** 1 Department of Pharmacy, The First Affiliated Hospital of Sun Yat-sen University, Guangzhou, Guangdong, China; 2 Department of Infection Prevention and Control, The Affiliated Guangdong Second Provincial General Hospital of Jinan University, Guangzhou, Guangdong, China; 3 Biosafety Laboratory, Zhongshan School of Medicine, Sun Yat-sen University, Guangzhou, Guangdong, China

**Keywords:** bloodstream infections, etiological diagnosis, Metagenomics Capture targeted next-generation sequencing, negative predictive value, pathogenic microorganisms

## Abstract

**Background:**

Bloodstream infections (BSIs) represent a significant public health concern. Metagenomic Capture targeted next-generation sequencing technology, as a newly emerging method for pathogen detection, has been applied in the etiological diagnosis of various infectious diseases and demonstrates good diagnostic efficacy. However, there is relatively limited research on the diagnostic value of this technology for the etiological diagnosis of BSIs.

**Methods:**

A comprehensive retrospective analysis was performed on patients suspected of having BSIs who were admitted to the Affiliated Guangdong Second Provincial General Hospital of Jinan University in 2024. These patients underwent both blood culture analysis and Metagenomic Capture targeted next-generation sequencing technology for diagnostic testing, and a detailed comparison of the results was conducted.

**Results:**

It was found that the Metagenomic Capture-targeted next-generation sequencing method has a shorter time to result [1.33 (1.18 - 1.69) vs 2.73 (1.89 - 3.84) days, *p* < 0.001], more pathogenic microbial species detected, higher positive detection rate and higher sensitivity than blood culture.

**Conclusions:**

Metagenomic Capture targeted next-generation sequencing technology is a promising tool for pathogen identification in BSIs, offering substantial methodological advantages in terms of turnaround time, detection breadth, and sensitivity. These diagnostic performance characteristics support its potential utility in clinical microbiology practice.

## Introduction

Bloodstream infections (BSIs) are a prevalent occurrence among inpatients and, in certain instances, can result in acute infections and sepsis, a systemic infectious response that can lead to circulatory failure, multiorgan dysfunction, and death (Buehler et al., 2016; Holmes et al., 2024). The annual incidence of BSIs has been estimated at 150 cases per 100,000 population, with a crude mortality rate of 17% defined as death within 30 days of a positive blood culture (Verway et al., 2022). The increasing rates of antimicrobial drug resistance (Antimicrobial Resistance Collaborators, 2022), the poor positivity of blood cultures for pathogenic microorganisms, and the technical limitations of blood microbiology testing have collectively transformed BSIs into a significant public health concern. The conventional approach to the cultivation of microorganisms involves the use of the blood culture method, which is then followed by the Gram staining technique. The test technician is responsible for providing a verbal report to the supervising clinician on the basis of the positive results of the culture. This verbal report includes the initial determination of the bacteria, as well as a follow-up antimicrobial susceptibility test (AST). The final report, which includes the AST can take 48 h or longer to obtain. The majority of these reports documented monomicrobial infections, with polymicrobial infections accounting for a minority of clinical cases, amounting to approximately 5-13% (Marra et al., 2011; Kontula et al., 2021).

Immediately following the collection of blood for culture, empirical and often broad-spectrum antimicrobial drug therapy is initiated in patients with suspected BSIs. Delays in microbial identification often result in failure to shift from broad-spectrum to targeted therapy in a timely manner. The rapid identification of BSI pathogens may have multiple potential benefits for patient management, including facilitating timely switching from empirical to targeted therapy. Blood cultures remain a cornerstone of routine etiological diagnostics (Lamy et al., 2020), yet their sensitivity remains suboptimal. While polymerase chain reaction (PCR)-based methods have been widely employed for the diagnosis of BSIs, their utility is often compromised by suboptimal sensitivity and specificity (Samuel, 2023). Although next-generation sequencing (NGS) techniques, particularly macrogenomic next-generation sequencing (mNGS), offer significant advantages in detecting pathogenic microorganisms, their application in serum-based diagnostics remains hampered by low positivity rates and significant cost constraints (Gu et al., 2019). Given these limitations, there is an urgent need for more sensitive, rapid, and cost-effective diagnostic methods to address the challenges of identifying pathogenic agents in BSIs. In this study, we systematically evaluated and characterized the etiologic performance of Metagenomics Capture (MetaCAP™) targeted next-generation sequencing (tNGS) as a potential diagnostic tool for identifying pathogens in BSIs.

## Materials and methods

**Figure 1:**
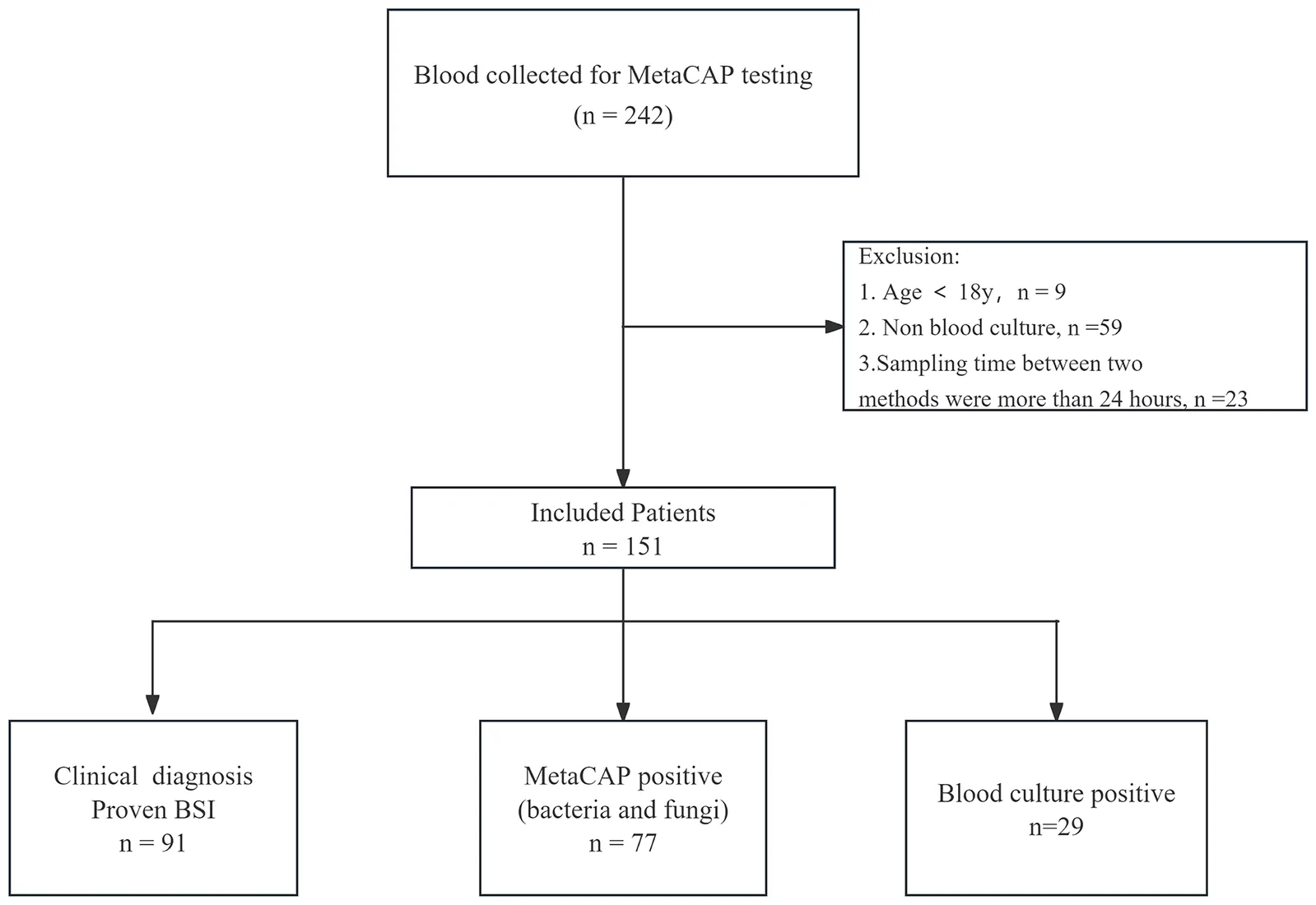
The schematic representation of the study profile.

In this study, we will compare blood cultures to explore the ability of high-throughput nucleic acid sequencing of MetaCAP™ pathogenic microorganisms to detect pathogens such as bacteria, fungi, viruses and others pathogens in BSIs. We will also compare the positive rates between high-throughput nucleic acid sequencing of MetaCAP™ pathogenic microorganisms and blood culture, aiming to provide accurate recommendations for sample selection in clinical testing of this technology. Comprehensive final clinical diagnostic will be taken as standards. Patients were included if they met the clinical criteria for suspected BSIs, underwent both blood culture and MetaCAP™ testing within 24 hours, and had complete clinical data available for review. Patients were excluded if they were < 18 years of age, had incomplete diagnostic testing or missing clinical data, or had blood culture results that were contaminated. A total of 151 patients were included in the final analysis. The study profile is depicted in **Figure 1**.

### Data collection

The research period for the specimens under investigation is from January 1, 2024 to December 31, 2024, at the Affiliated Guangdong Second Provincial General Hospital of Jinan University. It strictly adhered to ethical standards, in accordance with the principles of the Helsinki Declaration. Authors had not access to information that could identify individual participants during or after data collection. Information on blood culture specimens, including case numbers, genders, ages, specimen serial numbers, sampling times, report times, and blood culture results, was extracted from the hospital information system KR-Lis V5.0 (manufacturer: Kangrui Technology Co., Ltd., Guangdong Province, China). Information on blood specimens detected using high-throughput nucleic acid sequencing of MetaCAP™ pathogenic microorganisms, including case numbers, departments, names, genders, ages, specimen serial numbers, sampling times, report times, and detection results, was exported from the Jinyu Cloud Inspection System (manufacturer: Guangzhou Jinyu Medical Examination Center Co., Ltd., Guangdong Province, China). Information on patients with BSIs, including admission and discharge times, clinical diagnoses, biochemical (including C-reactive protein (CRP), Interleukin-6 (IL-6), Procalcitonin (PCT), White blood cells (WBC), Platelets, Red blood cells (RBC), Haemoglobin), catheterization status (respirators, catheters, and central venous catheters), and patient outcomes (death and discharge) were extracted from the electronic medical record system V6.0 (manufacturer: Beijing Jiahe Meikang Information Technology Co., Ltd.) and the hospital intelligent infection management system (source: Shanghai Lianxian Information Technology Co., Ltd.). The study was approved by the Ethics Committee of The Affiliated Guangdong Second Provincial General Hospital of Jinan University, Guangzhou, China (Approval No. 2025-KY-KZ-091-01). Due to the retrospective nature of the study and the use of a de-identified database, the Ethics Committee of The Affiliated Guangdong Second Provincial General Hospital of Jinan University waived the need of obtaining informed consent.

### Blood cultures

According to the procedure of bacteria and fungi culture in the microbiology laboratory of the Affiliated Guangdong Second Provincial General Hospital of Jinan University, blood culture was performed using the American BD-BACTE-CFX200 blood culture instrument with matching blood culture bottles, and adult patients were bilaterally double bottles (two aerobic bottles and two anaerobic bottles). Serum PCT was detected by using VIDAS PC automatic fluorescence immunoassay analyzer from Mérieux, France. The identification of bacteria was conducted using the BD Phoenix 100 automatic microbiology analyzer, manufactured in the United States, with the employment of bacterial identification reagents as supporting reagents.

### Metagenomics Capture targeted next-generation sequencing

Blood was collected in accordance with the collection procedures for blood cultures. A volume of 3 mL of blood was centrifuged at 1,600 rcf for 10 min at 4°C within 8 h of collection. The nucleic acids were extracted from 300 μL of plasma using the MasterPure™ DNA & RNA Extraction Kit (KS121-WSWTO-48, KingCreate, China), in accordance with the manufacturer’s instructions. Reverse de-hosting of nucleic acids is a technique used to isolate specific nucleic acids from a mixture. This can be achieved through differential cleavage for DNA and by using a probe to capture the human ribosome and then elute it for RNA. These methods are commonly used in the construction of libraries of nucleic acids. After the conversion of RNA to complementary DNA (cDNA), libraries were constructed through fragmentation, end repair, adapter ligation, and PCR amplification. Following this, eight uniquely barcoded libraries were combined for hybridization and captured using specific biotinylated probes for a duration of 2 hours. This process was carried out after library generation using the MetaCAP™ Pathogen Capture Metagenomic Assay Kit (KingCreate, China), in accordance with the manufacturer’s instructions. The enrichment panel focuses on capturing over 3000 important pathogens, with coverage of over 20,000 homologous pathogens. The detection range includes 1060 bacteria, 1402 viruses, 388 fungi, and 210 parasites. There are over 50 antibiotic resistance genes detected, with a total of 2627 genotypes. The detection limit for bacteria and fungi is as low as 50 CFU/mL, and for viruses it is as low as 50 copies/mL (Cai et al., 2024). The Qubit dsDNA HS Assay Kit (Thermo Fisher Scientific Inc., Waltham, MA, USA) was utilized to construct libraries of high quality. Sequencing was carried out on an Illumina MiniSeq platform with an average of 1 million reads per sample, set to 100-bp single end.

### Diagnostic performance analysis

The diagnostic results at the time of discharge for all subjects were utilized as the standard. Patients were included if they met the clinical criteria for suspected BSIs, including suspected bacteremia, fungemia, and secondary sepsis. The final clinical diagnosis at discharge, determined by an expert panel, served as the reference standard for evaluating the diagnostic performance of both testing methods. The expert panel consisted of two senior physicians, including one infectious disease specialist and one department director, both with extensive clinical experience. The final diagnosis was established through a comprehensive review of all available clinical data, including: (1) clinical signs and symptoms of infection; (2) laboratory findings (white blood cell count, C-reactive protein, procalcitonin, interleukin-6); (3) microbiological results from blood cultures and other relevant clinical specimens (sputum, urine, wound swabs, etc.); (4) imaging findings (chest X-ray, CT, ultrasound); (5) response to antimicrobial therapy; and (6) overall clinical course and outcome. For cases with polymicrobial results (e.g., bacteria + virus, bacteria + fungus, or virus + fungus), the expert panel adjudicated clinical significance based on the following criteria: (a) consistent clinical presentation compatible with the detected pathogens; (b) supportive laboratory and imaging findings; (c) appropriate clinical response to targeted antimicrobial, antifungal, or antiviral therapy; and (d) exclusion of colonization or contamination based on clinical context. Pathogens detected by MetaCAP™ alone without corresponding clinical evidence were considered clinically indeterminate and were not classified as true positives in the diagnostic performance analysis.

The sensitivity, specificity, positive predictive value (PPV), and negative predictive value (NPV) of the two different detection methods are calculated by taking the final clinical diagnosis as the reference standard. In order to facilitate comparison of the sensitivity, specificity, positive predictive value (PPV), and negative predictive value (NPV), it is important to note that high-throughput nucleic acid sequencing of MetaCAP™ pathogenic microorganisms method does not include viruses, protozoa, chlamydia, mycoplasma, rickettsia, and parasites.

### Statistical analyses

All statistical analyses were performed using SPSS statistical software, version 29.0. Categorical variables were compared using the chi-square test. Counted data were described by the number of cases (percentage), and the Kolmogorov-Smirnov test was used to verify the normality of the data. For continuous variables that followed a normal distribution, the mean ± standard deviation (SD) was used to describe them, and the differences between the groups were assessed using Student’s t test. Non-normally distributed data were expressed as the median and interquartile range (IQR, P25, P75), and non-parametric tests were analyzed using the Mann-Whitney U test. To identify independent risk factors for in-hospital mortality and to adjust for potential confounding variables, we performed multivariate logistic regression analysis. Those considered clinically relevant (age, sex, disease severity markers, and catheter utilization) were entered into a logistic regression model. All statistical analyses were evaluated at the statistical significance level of *p* < 0.05 (two-sided).

## Results

### Clinical characteristics of patients

The current investigation aimed to compare only those bacteria and fungi that were identified as positive through the MetaCAP™ method. The study included 151 patients, of whom 96 were male (63.58%). The length of hospitalization was found to be significantly longer in patients with a positive diagnosis than in those with a negative diagnosis, for both methods (*p* = 0.023 and *p* = 0.038). Among the clinical biochemical indices, for the MetaCAP™ method, the PCT was higher for pathogen-positive than pathogen-negative values, and the difference was statistically significant (2.50 (0.618 - 8.04) vs 0.96 (0.11 - 4.79), *p* = 0.006). Furthermore, platelet indices were lower in patients with positive pathogen detected than in patients with negative pathogen detected for both assays, and the difference was statistically significant (*p* = 0.006 and *p* = 0.022). In erythrocytes and hemoglobin, the presence of blood cultures was found to be higher in patients with a negative pathogen detection result than in those with a positive detection result. This difference was found to be statistically significant (*p* = 0.001 and *p* = 0.006). MetaCAP™ demonstrated a significantly quicker time to result in comparison to blood culture (1.33 (1.18 - 1.69) vs 2.73 (1.89 - 3.84) days, *p* < 0.001). The administration of pathogen delivery prior to the initiation of antimicrobial drug therapy led to a higher frequency of positive pathogen detection outcomes compared to negative pathogen detection outcomes for both tests, with a statistically significant difference (*p* < 0.001 and *p* = 0.004). The utilization rate of patients with positive pathogen detection for MetaCAP™ was significantly higher than that of patients with negative for all three devices: the ventilator, central venous catheter, and urinary catheter. This difference was statistically significant (*p* = 0.003). Similarly, central venous catheters and urinary catheters exhibited a higher utilization rate in patients with positive pathogen detection by blood cultures compared to those with negative pathogen detection, and this difference was also statistically significant (*p* = 0.075). In terms of patient prognosis, mortality was lower in patients with positive MetaCAP™ pathogen detection compared to those with negative pathogen detection (*p* = 0.003), while the difference between the rates of positive and negative blood culture pathogen detection was not statistically significant (*p* = 0.075) (**Table 1**). Multivariate logistic regression analysis was performed to identify MetaCAP™ positivity is not a significant independent predictor of mortality after adjusting for confounders (adjusted OR = 0.748, 95% CI: 0.177–3.165, *p* = 0.693). In the multivariate analysis, no conventional clinical baseline variables, including age, sex, procalcitonin, interleukin-6, platelet count, and indwelling catheter status, were identified as independent risk factors for in-hospital mortality.

**Table 1:** Clinical characteristics of patients.

Characteristics	MetaCAP™		Blood culture		*p*1-value	*p*2-value	*p*3-value
	Positive (n = 77)	Negative (n = 74)	Positive (n = 29)	Negative (n = 122)			
Age (mean ± SD)	60.35 ± 18.37	53.58 ± 17.73	63.31± 18.54	55.54 ± 18.02	0.023	0.04	0.4624
Male sex	50 (64.94)	46 (62.16)	17 (58.62)	79 (64.75)	0.723	0.537	0.549
Days of hospitalization	20.73 (11.75 - 38.67)	14.88 (6.92 - 29.17)	21.98 (11.75 - 45.77)	16.12 (7.68 - 30.96)	0.023	0.038	0.464
Time to result, day	1.33 (1.18 - 1.69)	–	2.73 (1.89 - 3.84)	–	–	–	< 0.001
Cultures prior to antibiotics	52 (67.53)	28 (37.84)	18 (62.07)	40 (32.79)	< 0.001	0.004	0.596
Number of deaths	6 (7.79)	19 (25.68)	8 (27.59)	17 (13.93)	0.003	0.075	0.007
Blood Parameters – median (interquartile range)							
Interleukin-6, pg/mL	58.22 (25.18 - 250.10)	71.06 (27.11 - 319.90)	55.68 (24.57 - 226.00)	70.76 (26.76 - 316.80)	0.692	0.89	0.829
Procalcitonin, ng/mL	2.50 (0.618 - 8.04)	0.96 (0.11 - 4.79)	1.94 (0.48 - 15.46)	1.45 (0.28 - 5.61)	0.006	0.326	0.804
White blood cells,×10^9^/L	9.79 (5.26 - 16.47)	9.69 (4.93 - 14.26)	7.91 (3.41 - 12.38)	10.07 (5.86 - 15.94)	0.756	0.121	0.184
Platelets, ×10^9^/L	135.50 (54.25 - 268.00)	173.00 (49.50 - 247.30)	99.00 (26.00 - 205.50)	155.50 (60.00 - 278.80)	0.006	0.022	0.428
Red blood cells, ×10^12^/L	2.63 (2.09 - 3.58)	3.29 (2.29 - 3.99)	2.13 (1.91 - 2.95)	3.07 (2.34 - 3.77)	0.057	0.001	0.061
Haemoglobin, g/L	76.00 (61.25 - 104.50)	87.50 (65.00 - 109.50)	64.00 (57.25 - 89.00)	86.50 (65.00 - 108.00)	0.138	0.006	0.091
C-reactive protein, mg/L	71.10 (38.00 - 237.30)	90.00 (42.15 - 215.40)	72.52 (51.48 - 236.00)	78.65 (37.55 - 217.60)	0.851	0.879	0.922
Indwelling catheter							
Endotracheal tube	29 (37.66)	14 (18.92)	8 (27.59)	35 (28.69)	0.011	0.906	0.332
Central venous catheter	55 (71.42)	32 (43.24)	20 (68.97)	47 (38.52)	< 0.001	0.003	0.804
Urethral catheter	54 (70.13)	23 (31.08)	18 (62.07)	39 (31.97)	< 0.001	0.003	0.428

Data are expressed as n (%) or median (interquartile range), unless otherwise stated. The *p*1-value compares positive and negative outcomes via the MetaCAP™ methodology, while the *p*2-value assesses the blood culture results. The *p*3-value contrasts positive results from two methods.

### Comparison of detection rates of pathogens between two testing methods

If the proportion of patients with at least one positive pathogen detected, including bacteria, fungi, viruses, chlamydia, mycoplasma, and others, in any sample is considered the overall positivity rate, then the overall positivity rate is 85.43% (129/151), with an 85.43% positivity rate in the MetaCAP™ method, which is higher (*p* < 0.001) than the positivity rate of blood cultures at 19.21% (**Table 2**). If only the detection rates of bacteria and fungi are compared, the overall positivity rate is 52.98% (80/151), with a MetaCAP™ positivity rate of 50.99%, which is also higher (*p* < 0.001) than the blood culture positivity rate of 19.21% (**Table 3**).

**Table 2:** Comparison of positive detection rates of pathogens between MetaCAP™ and blood cultures.

Blood cultures	MetaCAP™		Total	*p* - value
	Positive	Negative		
Positive	29	0	29	
Negative	100	22	122	
Total	129	22	151	< 0.001

**Table 3:** Comparison of positive detection rates of pathogens between MetaCAP™ (bacteria and fungi) and blood cultures.

Blood cultures	MetaCAP™ (bacteria and fungi)		Total	*p* - value
	Positive	Negative		
Positive	26	3	29	
Negative	51	71	122	
Total	77	74	151	< 0.001

### Comparison of sensitivity and specificity between two testing methods

**Figure 2:**
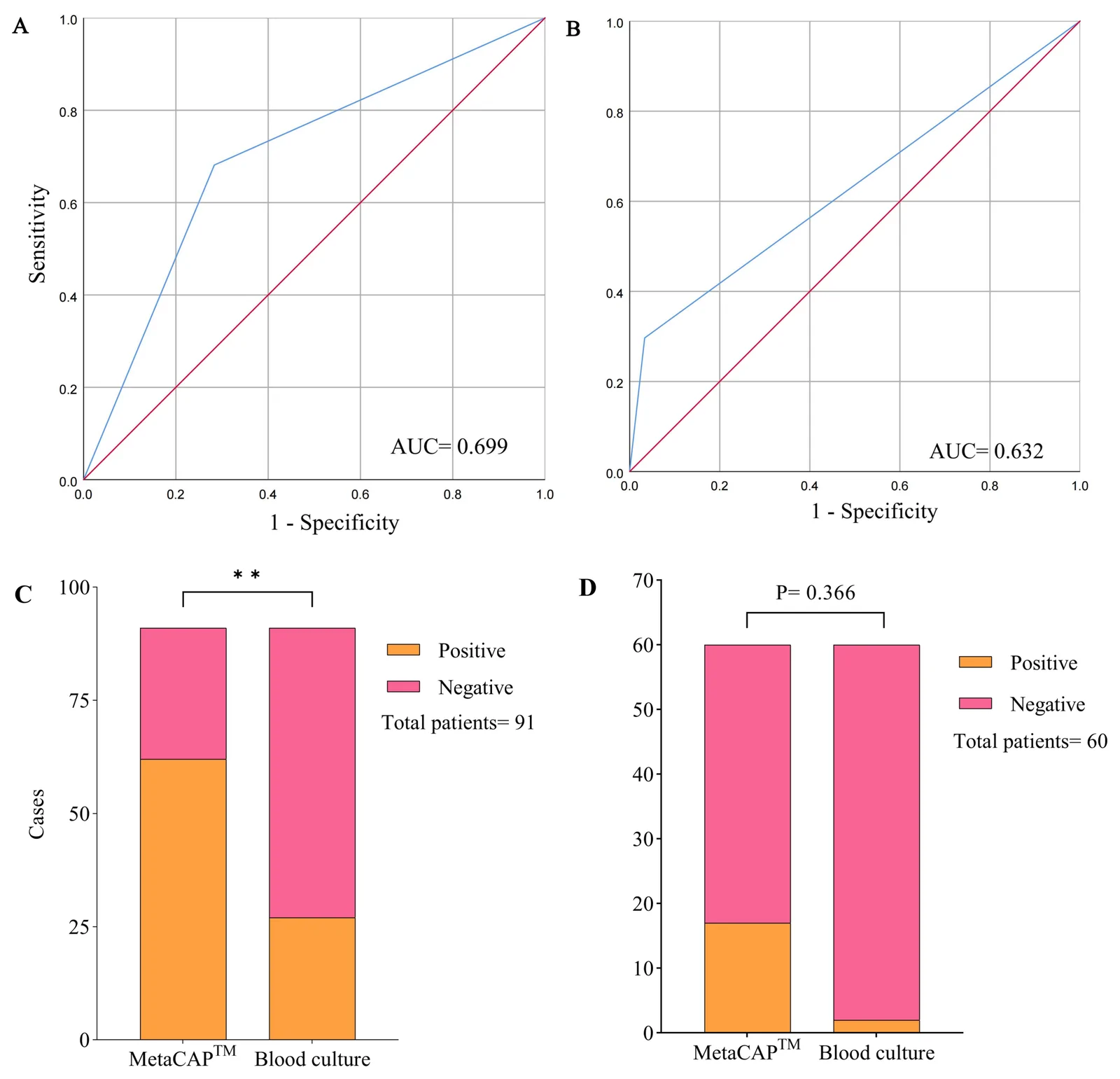
Sensitivity and specificity between two testing methods. **(A)** Receiver Operating Characteristics Curves for Diagnostic and Clinical Diagnostic MetaCAP™ Methods. **(B)** Receiver Operating Characteristics Curves for Blood Culture Diagnosis and Clinical Diagnosis. **(C)** Comparison of the sensitivity of blood culture diagnosis and MetaCAP™ diagnosis. **(D)** Comparison of the specificity of blood culture diagnosis and MetaCAP™ diagnosis. *p* - value derived from chi-square test between the two methods. Symbols for P values: *<.05, **<.001.

The final diagnostic results at the time of discharge for all subjects were used as the standard to compare the sensitivity and specificity of MetaCAP™ (for bacteria and fungi) and blood cultures, with MetaCAP™ showing a sensitivity of 68.13%, a positive predictive value of 78.48%, a specificity of 71.66%, a negative predictive value of 59.72%, and the Receiver Operating Characteristics (ROC) Curve was plotted, with area under curve (AUC) being 0.699, *p* < 0.001. The blood culture had a sensitivity of 29.67%, a positive predictive value of 93.10%, a specificity of 96.67%, a negative predictive value of 47.54%, and AUC was 0.632, *p* = 0.006 (**Figures 2A, B**). In the comparison of sensitivity and specificity between MetaCAP™ and blood cultures, MetaCAP™ had a higher sensitivity (68.13%) than blood cultures (29.67%)(*p* < 0.006), while there was no significant difference (*p* = 0.366) in specificity (71.66% vs 96.67%)(**Figures 2C, D**).

### Distribution of detected pathogenic microorganism

**Figure 3:**
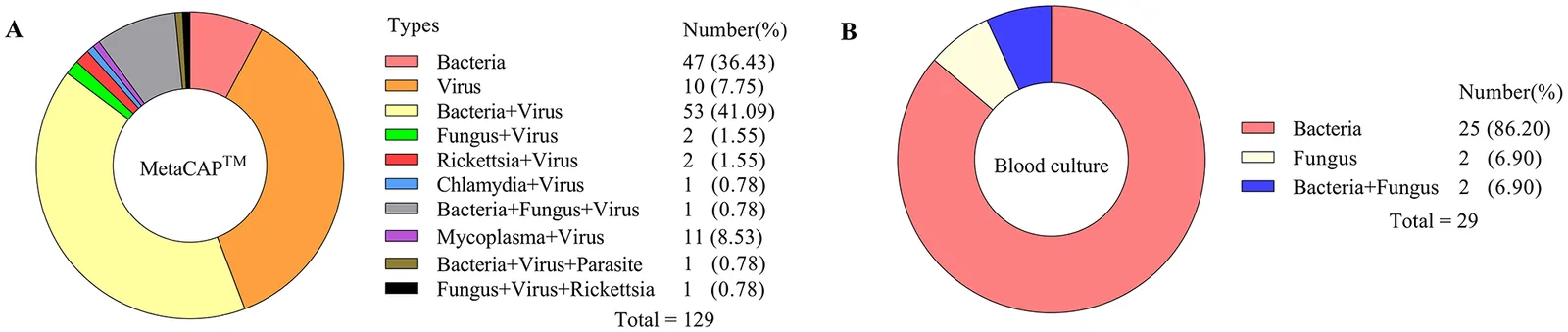
**(A)** Pathogenetic classifications detected by the MetaCAP™ method. **(B)** Classification of pathogenicity detected by blood culture methods.

**Figure 4:**
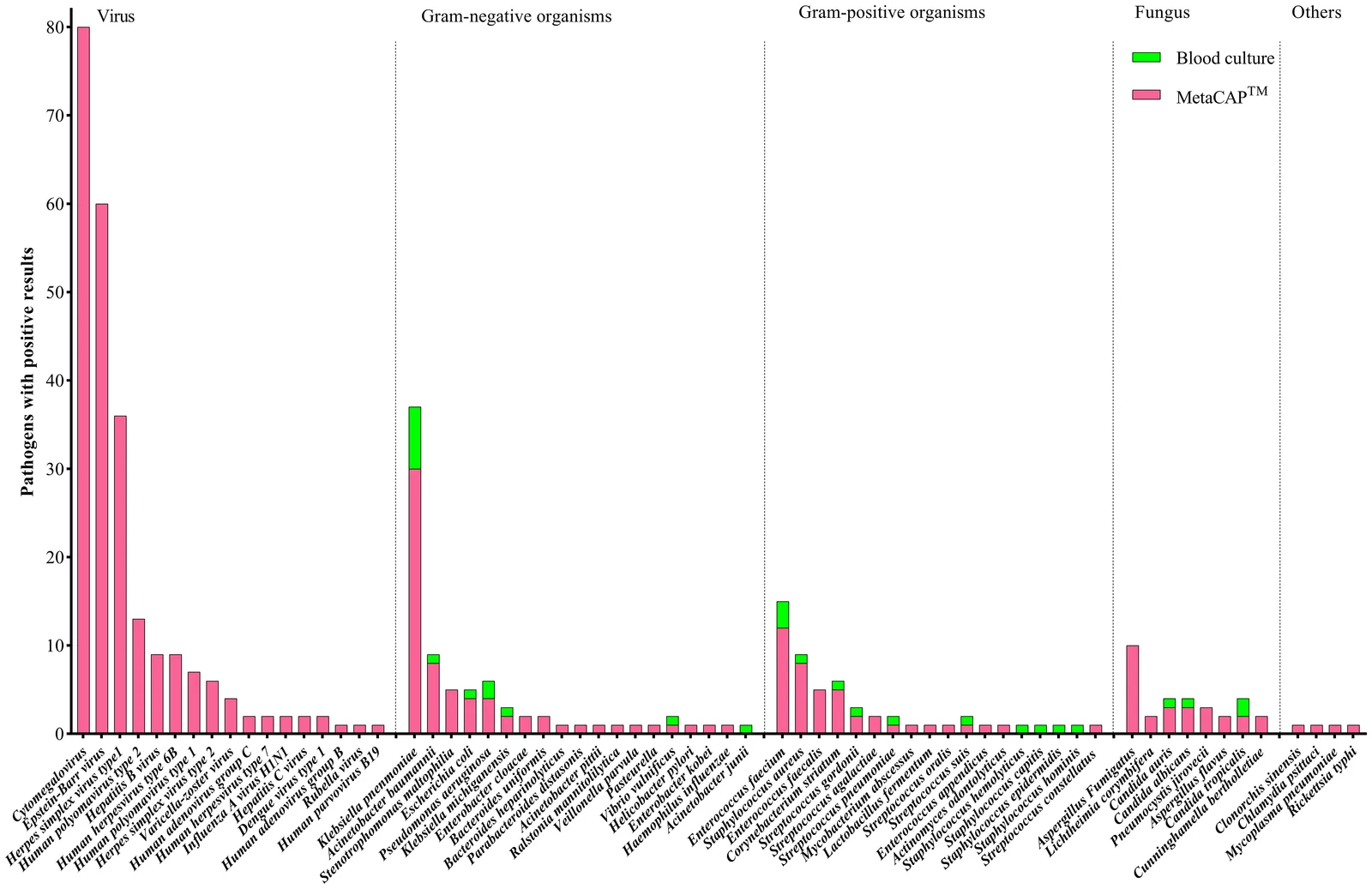
Distribution of detected pathogenic microorganism of MetaCAP™ and blood culture in suspected bloodstream infections patients.

In the detection and classification of pathogens, MetaCAP™ detected a higher number of positive results for Bacteria + Virus (53 cases), Virus (47 cases), Bacteria + Fungus + Virus (10 cases), and Bacteria (10 cases). It was also able to detect *Mycoplasma*, *Chlamydia*, *Rickettsia*, and parasites. The specific results were *Rickettsia* + Virus (2 cases), *Chlamydia* + Virus (1 case), *Mycoplasma* + Virus (1 case), Bacteria + Virus + Parasite (1 case), and Fungus + Virus + *Rickettsia* (1 case) (**Figure 3**). Blood cultures mainly detected bacteria, and the detection of viruses, *Mycoplasma*, *Chlamydia*, *Rickettsia*, and parasites was limited by the detection technology and could not be detected. The specific detection numbers are shown in **Figure 4**.

## Discussion

The results demonstrated that the MetaCAP™ method offers several methodological advantages, including a significantly shorter turnaround time, a broader spectrum of detectable pathogens, a higher positive detection rate, and superior sensitivity compared to blood culture.

The patient outcome is significantly influenced by delayed therapy, and the rapid and accurate identification of pathogens is crucial for the effective care of patients (Lamy et al., 2020). The standard time to result for a positive blood culture typically ranges from 2 to 4 days, a duration that is undeniably protracted and not conducive to effective acute patient management (Idelevich et al., 2019). In the present study, it was established that the time required for the results of Metagenomics Capture targeted next-generation sequencing to be produced was reduced by half. This improved turnaround time may facilitate more timely and informed antimicrobial prescribing decisions by the clinician-in-charge, representing a meaningful advantage in the management of patients with suspected BSIs (Blauwkamp et al., 2019). In addition, to further increase the rate of pathogen detection, blood specimens should be sent for testing prior to antimicrobial therapy (Garcia et al., 2015).

Post-2004, *Escherichia coli* surged to prominence as the predominant pathogen responsible for BSIs, commanding a significant share of 20 to 27 percent of all BSIs, while *Staphylococcus aureus* maintained a substantial presence with approximately 19 to 25 percent of the cases (Diekema et al., 2019; Kontula et al., 2021; Verway et al., 2022). However, in our study, the detection of pathogenic bacteria was more prevalent with *Klebsiella pneumoniae*, *Enterococcus faecalis*, and *Staphylococcus aureus*, which may be closely related to BSIs, pulmonary infections, urinary tract infections, surgical incisions, catheter placements, and other such conditions (Rao et al., 2021). Blood cultures are collected from venipuncture through the skin or from indwelling catheters, both of which are sites of bacterial colonization (Kaasch et al., 2014). Blood cultures are the gold standard for the diagnosis of the causative agents of BSIs, but they have notable limitations such as low sensitivity and specificity and a long time to diagnosis (Wilson, 2020). Moreover, the presence of multiple pathogens in the same positive blood culture vial may complicate the use of rapid drug sensitivity test methods (Lutgring et al., 2018). Fungi have become increasingly prevalent as isolates in BSIs, their incidence having notably risen over the past few decades (Pfaller et al., 2019).

Compared to blood cultures, MetaCAP™ methods are not only capable of detecting bacteria and fungi, but also viruses, *rickettsia*, *chlamydia*, *mycoplasma*, and parasites, enabling the detection of some rare pathogens, such as *Leptospira (*Wilson et al., 2014), *Pneumocystis (*Zhang et al., 2019), *Legionella (*Huang et al., 2019). The MetaCAP™ methods also have a significant advantage in terms of opportunistic pathogens and mixed infections (Liu et al., 2022). In this study, the MetaCAP™ methods detected cytomegalovirus, Epstein-Barr virus, and herpesvirus, highlighting the potential of the MetaCAP™ methods as a supplementary tool for pathogen detection in clinical practice. Research has shown that viral infection is an independent risk factor associated with sepsis-related death (Ong et al., 2017). Blood cultures fail to culture viruses, *mycoplasmas*, *chlamydiae*, *rickettsiae*, and parasites. The ability of MetaCAP™ methods to do so enables timely and accurate diagnosis, which can precisely provide antimicrobial treatment, ultimately improving the prognosis of patients with BSIs. Additionally, some critically ill patients may not only have a single bacterial infection but could also be co-infected with other pathogens such as viruses, accounting for 6%-34% of BSIs involving mixed infections (Lin et al., 2010). Compared to patients with BSIs caused by a single microorganism, those with multiple mixed microbial infections may not be adequately treated with a single antibiotic, especially when fungal and viral infections are involved.

Although our study has confirmed the superiority of Metagenomics Capture targeted next-generation sequencing, there are still some limitations to this experimental method. First, although we adjusted for a range of clinically relevant covariates in the multivariate logistic regression analysis, we were unable to incorporate critical severity of illness scores, such as the SOFA or APACHE II score at admission, or comorbidity indices such as the Charlson Comorbidity Index. Despite our proactive efforts to retrieve these data from the hospital medical records system, we encountered difficulties related to institutional ethics requirements and incomplete documentation for a substantial proportion of patients, which ultimately precluded their inclusion in the analysis. The absence of these important baseline parameters may have resulted in residual confounding, and the observed mortality difference should therefore be interpreted with caution. Second, the relatively small sample size may have limited the statistical power to detect modest but clinically meaningful associations, and it also precluded meaningful pathogen stratified subgroup analyses, particularly for fungal infections and mixed infections, which had particularly small case numbers. Third, the strategy of aggregating all pathogen positive results together for the primary analysis, while necessitated by sample size constraints, may have obscured pathogen specific differences in mortality risk. Finally, this was a single center observational study, which may restrict the generalizability of our findings to other settings. Future multicenter studies with larger cohorts and comprehensive collection of severity, comorbidity, and pathogen stratified data are warranted to validate and extend our results.

## Conclusion

The methodological strengths of this approach, particularly its substantially shorter turnaround time, broader detection spectrum, and higher sensitivity relative to conventional blood culture, make it a promising candidate for pathogen identification in BSIs. As the MetaCAP™ workflow becomes increasingly streamlined and more cost-effective, it is expected to serve as a valuable supplementary tool in clinical microbiology practice.

## Data Availability

The original contributions presented in the study are included in the article/supplementary material. Further inquiries can be directed to the corresponding author.
